# Surface-Enhanced Absorption Spectroscopy for Optical Fiber Sensing

**DOI:** 10.3390/ma13010034

**Published:** 2019-12-19

**Authors:** Silje S. Fuglerud, Karolina Milenko, Astrid Aksnes, Dag R. Hjelme

**Affiliations:** 1 Department of Electronic Systems, Norwegian University of Science and Technology, O.S. Bragstads plass 2b, 7034 Trondheim, Norway; 2 Department of Endocrinology, St. Olavs University Hospital, Prinsesse Kristinas gate 3, 7030 Trondheim, Norway

**Keywords:** SEIRAS, Vis/NIR spectroscopy, optical fiber, sensing, surface enhancement, gold

## Abstract

Visible and near-infrared spectroscopy are widely used for sensing applications but suffer from poor signal-to-noise ratios for the detection of compounds with low concentrations. Enhancement by surface plasmon resonance is a popular technique that can be utilized to increase the signal of absorption spectroscopy due to the increased near-field created close to the plasmons. Despite interest in surface-enhanced infrared absorption spectroscopy (SEIRAS), the method is usually applied in lab setups rather than real-life sensing situations. This study aimed to achieve enhanced absorption from plasmons on a fiber-optic probe and thus move closer to applications of SEIRAS. A tapered coreless fiber coated with a 100 nm Au film supported signal enhancement at visible wavelengths. An increase in absorption was shown for two dyes spanning concentrations from 5 × 10^−8^ mol/L to 8 × 10^−4^ mol/L: Rhodamine 6G and Crystal Violet. In the presence of the Au film, the absorbance signal was 2–3 times higher than from an identically tapered uncoated fiber. The results confirm that the concept of SEIRAS can be implemented on an optical fiber probe, enabling enhanced signal detection in remote sensing applications.

## 1. Introduction

Visible (Vis) and near-infrared (NIR) spectroscopy, as applied to the detection of biomolecules and the determination of the constituents of unknown substances, both have the advantage of little or no sample preparation as well as fast data acquisition times [1,2]. A variety of fields employ spectroscopy—such as health, pharmacy, safety, food manufacturing, and forensic sciences—to identify and quantify substances [3]. When combined with a suitable optical fiber probe interface [4], the use of spectroscopy can extend to remote sensing in new applications. However, Vis/NIR absorption spectroscopy normally measures overtones of the molecule’s vibrational bands; the absorption cross-section is therefore much weaker than that of the mid-infrared (MIR) (i.e., on the order of 10^−22^ cm^2^ for overtones and combination bands compared to $10^{-18}$ cm^2^ for fundamental vibrations), limiting the sensitivity in the Vis/NIR range. Due to the broad availability and lower cost of detectors, sources, and fibers in the Vis/NIR range, however, it is still worth exploring. Here, we aim to apply plasmonic surface enhancement, as is widely applied in surface-enhanced Raman spectroscopy (SERS) and plasmon-enhanced fluorescence (PEF), to enhance the absorption signal of Vis/NIR spectroscopy. The implementation of surface-enhanced infrared absorption spectroscopy on fibers for NIR spectroscopy could expand the aforementioned areas of use: for example, in online fiber-based monitoring in food science [5]. Similarly, this method could increase the signal-to-noise ratio (SNR) when measuring glucose with an optical probe and thus enable the accurate detection of physiological levels of glucose with a fiber probe for the treatment of diabetes.

Three types of interactions between plasmons and molecules have been described: (1) a plasmon resonance wavelength shift caused by adsorbed molecules without absorption, (2) enhanced absorption due to an increased near-field if the molecules exhibit absorption close to the plasmon resonance wavelength, and (3) fluorescence enhancement [6]. SERS utilizes the second mechanism twice, both in absorption and the Raman scattering that follows. PEF utilizes both the second and the third mechanism, as both the absorption and the following emission can be enhanced due to the plasmon–molecule interaction [7,8]. The plasmon–molecule interaction may also be exploited to enhance the absorption signal in classical absorption spectroscopy. First, shown by Hartstein [9], surface-enhanced infrared absorption spectroscopy (SEIRAS) utilizes metal nanostructures to produce an enhancement effect for infrared spectroscopy; several subsequent works have investigated this effect with a focus on tailoring the resonance wavelength with different nanostructures and antennas [10,11,12,13]. More recently, Fano resonances were discovered and explained with respect to narrow molecular vibrations [14]. The plasmonic response at different stages of growth from Au nanoislands to films has also been investigated for an attenuated total reflection (ATR) SEIRAS probe [10]. Some works have demonstrated the importance of the film/semi-film growth parameters, such as thickness and deposition rate, for the plasmon characteristics [15,16]. However, the effect of SEIRAS has mostly been shown in lab conditions that are not easily adapted to optical fibers using the Kretschmann configuration with a prism or using an ATR probe [9,10,13,15]. For practical applicability, we here implement the enhancement directly on an optical fiber.

Numerous studies exist on the topic of the first plasmon–molecule mechanism (surface plasmon refractive index-based sensing), which is also adapted to fiber sensing. In this method of surface plasmon resonance (SPR) sensing, the plasmon resonance wavelength is used to determine changes in the refractive index and, therefore, the analyte presence [6,17,18,19,20,21]. Although this technique can be highly sensitive to refractive index changes, we are here interested in complex aqueous mixtures where the refractive index will be influenced by processes unrelated to the analyte of interest. Functionalization of the surface with a binding agent tailored to the analyte can be used to overcome this problem but is not always an option. Although many approaches exist for immobilization of biomolecules on metal surfaces, challenges related to the binding in question persist, including: high cost and labor-intensive preparation, specificity, and denaturation over time (making it less applicable to continuous sensing) [22]. Absorption spectroscopy, on the other hand, does not require any specific binding but instead detects the changes in the optical absorption of the molecules in question. Higher sensitivities might be obtained with SPR combined with the right chemical bonding scheme, but might not be feasible due to the cost and/or denaturation of the chemicals in the environment of interest. For this and other such situations, the generality of absorption spectroscopy can be preferable.

In this paper, we aimed to obtain surface-enhanced absorption from plasmons on a fiber-optic probe and thereby come closer to more widespread applications of SEIRAS. We extend the knowledge from previous work on SPR sensing on optical fibers and use this to implement surface-enhanced absorption spectroscopy on a fiber probe using commonly available optical fibers in the visible range. We first present the underlying theory and mechanism of surface-enhanced absorption spectroscopy and further describe the characterization and design of the Au layer for surface enhancement. We demonstrate the enhancement present on the fiber with two dyes and discuss the results and conclusions.

## 2. Theory

Beer–Lambert’s law provides a description for optical fiber evanescent field-sensing in fluids. It states that the absorbance of a sample in one mode is a function of the sample constituent concentrations,

(1) $$A=\sum_{i=1}^{N}\varepsilon_{i}c_{i}\eta_{p}l,$$

where *N* is the number of different constituents of the sample, $\varepsilon_{i}$ is the molar absorptivity of the *i*th analyte, $c_{i}$ is the concentration of the *i*th analyte, and *l* is the path length through the sample. We have omitted here the additive scattering $\mu_{i}$, which is often negligible. The constant $\eta_{p}$ accounts for the power distribution within the evanescent field [23]. Beer–Lambert’s law is normally linear with increasing analyte concentration, however, the power distribution $\eta_{p}$ may not be constant in an evanescent field excited by an multi mode (MM) fiber. Each mode in the initial distribution experiences different absorption, resulting in an altered modal distribution and, therefore, a unique response can be expected based on the fiber design [23]. The absorption coefficient is defined as $\alpha =\sum_{i=1}^{N}\varepsilon_{i}c_{i}$. At a given path length *l*, the transmitted intensity is $I\left(\lambda \right)=I_{0}\left(\lambda \right)\exp\left[-A(\lambda )\right]$, where $I_{0}\left(\lambda \right)$ is the reference intensity [24] (p. 10). The absorbance can therefore be determined experimentally from

(2) $$A=-\ln\left(\frac{I}{I_{0}}\right).$$

Enhancement occurs in the presence of surface plasmons, which can be excited on the surface of a metal film (SPR) or particle (localized surface plasmon resonance (LSPR)) and propagate along the surface of the film or oscillate around the particle. The Kretschmann configuration is a common excitation geometry for SPRs, where the plasmon wave is excited with light at incident angle θ through a prism with a thin metal film on top [17,25]. The dispersion relation for the surface plasmon at such a semi-infinite surface is

(3) $$k_{SP}=k_{0}{\left(\frac{\varepsilon_{m}\varepsilon_{d}}{\varepsilon_{m}+\varepsilon_{d}}\right)}^{1/2},$$

where $\varepsilon_{m}$ and $\varepsilon_{d}$ are, respectively, the complex permittivity of the metal and the complex permittivity of the dielectric in which the surface wave propagates, while $k_{SP}$ and $k_{0}$ are the wave vectors of the surface plasmon and incident light [26]. This relation imposes a condition on the incident light to match the real part of the wave vector of the surface plasmon:

(4) $$k_{x}=k_{0}n_{p}\sin\theta ,$$

where $n_{p}$ is the refractive index of the medium where the light propagates (a prism in the Kretschmann configuration) and θ is the angle of the incoming light with the metal surface normal [17,18,24].

In this paper, a tapered optical fiber replaces the Kretschmann configuration for miniaturization and provides more flexible sensing. Optical fibers have previously been successfully utilized for SPR sensing [17,19,21]. An approximation of the number of modes in an MM fiber is

(5) $$M\approx \frac{{\left(\frac{2\pi }{\lambda_{0}}a\mathrm{NA}\right)}^{2}}{2},$$

where $\lambda_{0}$ is the vacuum wavelength, *a* is the radius of the fiber core, and NA is the numerical aperture [27]. For an MM fiber with NA=0.22, 2a=105μm and λ=532 nm, we find M≈9300. For a coreless fiber piece excited by such an MM fiber, a full ensemble of modes propagating with different angles will be available to excite the plasmons. Incoming light with a range of angles θ can fulfill the condition in Equation (4), which would lead to a spectrally broad plasmon resonance peak observed in transmission. The plasmon resonance wavelength is determined by the material parameters, the metal film thickness, and the excitation light angle θ.

In a design based on metal particles instead of a metal film, the plasmon resonance peak is given by the LSPR. The penetration depth of the field generated around the LSPR is on the order of tens of nm, while an SPR wave has a penetration depth of a few 100 nm [8,26,28,29] depending on the material parameters. The experimental requirements of LSPR can be more agreeable, as the condition in Equation (4) does not need to be fulfilled. The size, shape, and pattern of the metal particles in an LSPR design determine the resonance wavelengths and the strength and shape of the electrical field [30]. LSPR is used to tailor the plasmon resonance wavelength to the application [12,13]. For SEIRAS, islands or quasi-films have typically provided better signal enhancement than a metal film [3,10,30].

The surface-enhanced effect for absorption spectroscopy is often explained by both electromagnetic and chemical contributions. In the framework of SEIRAS, the electromagnetic effect arises from an increased near-field that accompanies plasmonic excitation. The electromagnetic effect is divided into resonant and non-resonant: resonant SEIRAS is obtained when sharp LSPRs are resonantly tuned to the infrared vibration in question, while non-resonant SEIRAS has overlapping plasmon and vibrational peaks that are not resonantly coupled [3]. Since we are investigating dyes, the mechanism in question is electronic excitation of the dye from the ground state to the first excited electronic state rather than a vibrational state. A similar effect to resonant/non-resonant SEIRAS of weak and strong coupling to dyes has been reported [7]. The primary technique that utilizes plasmon-enhancement in dyes has been the PEF [6,31], where the enhancement can occur both as the light is absorbed, and as the light is emitted as fluorescence [7]. In this study, the only measurable effect is the absorption enhancement of the dyes, as the subsequent fluorescence has no way to couple to the guided fiber modes.

## 3. Materials and Methods

### 3.1. Fiber Preparation

**Figure 1:**
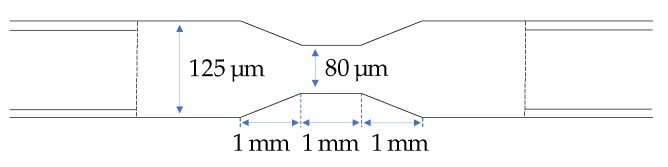
Dimensions of the tapered coreless fiber.

Cleaved 2 to 5 long 125 μm diameter coreless termination fibers (Thorlabs (Newton, NJ, USA)) were spliced between two bare MM fibers (Thorlabs) with 105 μm core and 125 μm cladding using a Fujikura FSM-100P fusion splicer (Tokyo, Japan). To obtain a more favorable modal distribution in the sensing region, the coreless part of the fiber was tapered by simultaneously softening the glass by an arc and pulling the fiber to 80 μm using the fusion splicer. The resulting dimensions were 1 mm taper length and 1 mm taper-down length on each side, as shown in Figure 1. The taper was found to give higher signal absorption values when compared to untapered pieces experimentally. A possible reason for this is that the transition from the MM fiber to the coreless fiber has a relatively low numerical aperture (lower than the fiber NA, which refers to air) which does not excite higher-order modes to a large degree in the coreless fiber. Although the tapering region is relatively long, it is nonadiabatic due to the closely spaced modes. Therefore, the taper will induce mode coupling and excite higher modes.

**Figure 2:**
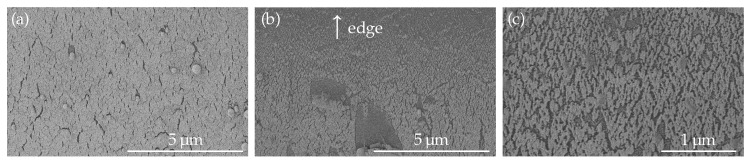
SEM images of the deposited 100 nm Au film. (**a**) the center of the film is almost continuous; (**b**) the film thins towards the edge of the deposition layer; (**c**) a closer look at the thinner part of the film towards the edge.

The tapered fibers were coated with Au in an AJA Sputter and Evaporator (Scituate, MA, USA at NTNU, NorFab) with a deposition rate of 5 Å/s, with thicknesses varying from 5 nm to 100 nm on one side. This resulted in a continuous gold film on top of the fiber that became thinner and transitioned to gold islands as the fiber surface curved. A semi-film with randomly arranged islands was deposited towards the edge of the film as the fiber curved, as seen with the SEM Apreo (ThermoFisher Scientific (Waltham, MA, USA) at NTNU, NorFab) and shown in Figure 2. As previously reported [10,16], a semi-film with gaps that allows for in-gap hot spots is ideal for the enhancement provided by SEIRAS. Thus, the imperfection of the film deposited on the fiber is beneficial for field enhancement. For the final absorption measurements, an Au thickness of 100 nm was selected.

### 3.2. Absorption Spectroscopy Measurements

**Figure 3:**
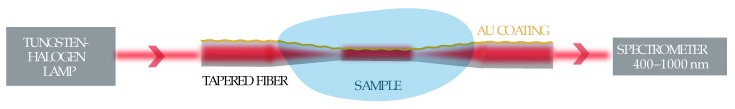
Experimental setup for measuring aqueous samples.

After Au film deposition, the tapered fibers were spliced to pigtailed MM fiber patch cables with the same dimensions as the previously used MM fibers for optimal connection. A broadband halogen lamp (SLS201L, Thorlabs) was connected to one end and illuminated the fiber. The taper and the connected fiber were immersed in an aqueous sample by applying a droplet of the sample on top of the fiber. The change in absorption in the evanescent field was measured with an OceanOptics USB2000 grating spectrometer (Largo, FL, USA) detecting wavelengths from 400 to 1000 nm. The setup is illustrated in Figure 3.

The samples used were dyes with a high absorption cross-section in the visible spectrum (on the order of $10^{-17}$ cm^2^): Rhodamine 6G (R6G, Sigma-Aldrich (St. Louis, MO, USA)) and Crystal Violet (CV, Sigma-Aldrich). Both powders were dissolved in ultrapure (Merck Millipore (Kenilworth, NJ, USA)) water and diluted to different concentrations from 5 × 10^−8^ mol/L to 8 × 10^−4^ mol/L. Prior to any sample measurement, the fiber was measured in air. The condition in Equation (3) is not met for the wavelengths in question in air; therefore, absorption peaks are absent and these measurements were used as a transmission reference. After a measurement, the exposed area was repeatedly flushed with water and ethanol (one to five times) until the signal returned to the baseline water measurement. Water was measured frequently for reference.

In the analysis, the dark noise spectrum was subtracted from the raw sample measurements. All single raw sample measurements were then referenced to the preceding air measurement through Equation (2). All measurements were averaged with an eight-point moving mean and a Savitzky–Golay filter of 3rd order and a frame length of 11. The average background spectrum of water for the specific tapered fiber was subtracted from the absorption measurements of the dyes. Instead of mean-centering, the spectra were centered based on the wavelengths above 700 nm, as the absorbance at these wavelengths was consistently low for all of the analytes used.

## 4. Results

### 4.1. Ideal Au Thickness

**Figure 4:**
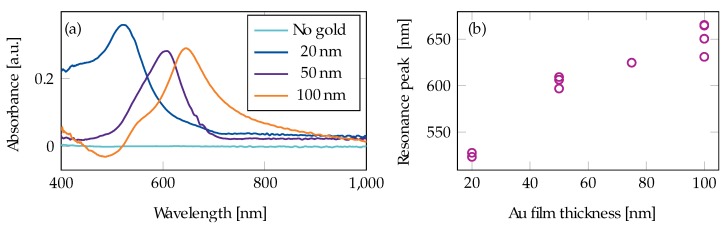
(**a**) examples of the characterization of a resonance wavelength measured in water with air as reference for 20 nm, 50 nm, and 100 nm Au films. The spectrum from a tapered fiber without an Au film is plotted for comparison; (**b**) resonance wavelength as a function of Au thickness.

Au thicknesses in the range 5 nm to 100 nm were investigated, both on tapered and untapered coreless fibers. However, the resonance peak of the 5 nm films was ambiguous and, as such, will not be included in the analysis. The resonance wavelength materialized as a distinct absorption peak in the spectrum and was found by immersion in water with air as a reference (in Equation (2)). The water absorption is less than 0.1 mm ^−1^ for the wavelengths in question and the contribution to the measured absorbance is therefore negligible. Representative samples of the absorbance in water and, thereby, the absorption due to the Au film is shown in Figure 4a for Au films with thickness 20 nm, 50 nm, and 100 nm on both tapered and untapered fibers. An example of water measured on an uncoated fiber is also included for reference. There was no significant difference between the peak position on untapered and tapered fibers. The difference in the intensity of the absorption peaks of the Au films in Figure 4a is representative of experimental variation between samples. The average peak absorption wavelength for a given coated fiber as a function of thickness is plotted in Figure 4b. For some Au thicknesses, several fibers were manufactured, showing some variance in the resonance peak with the same protocol. Across all samples, the peak absorption wavelength increased with increasing film thickness.

A possible explanation for the red-shift seen in the resonance wavelength with increasing film thickness is that the resonance wavelength is influenced by the structure on the side of the fiber, which resembles a bi-continuous structure reported in a previous LSPR study [32]. When transitioning from separated nano-particles to islands and nano-films for gold and silver, a similar trend to the results presented in Figure 4b appears [32,33]. With longer deposition times, the metal layer becomes more continuous, leading to a larger red-shift. This effect is due to the size distribution of the metal particles. It is worth noting that an SPR wave is only excited by light with Transverse Magnetic (TM)-polarization [24] (p. 27) in relation to the film. In this experimental setup, the incoming light does not harbor a specific polarization, and we can thus assume that only half of the light can excite an SPR. For LSPR on the other hand, light of any polarization can couple to the resonance. Another possible explanation for the red-shift of the resonance peak is that the effective refractive index of the plasmon mode decreases with thickness for an asymmetric plasmon mode in a metal film between two dielectrics [26] (p. 16). A lower effective refractive index gives a lower frequency for the phase-matching to the optical fiber mode with a fixed angle of incidence.

Experimentally, we found that a direct spectral overlap of the resonance peak with the absorption peak of the sample did not give as much signal enhancement as a partial overlap. We presume that this reflects that the in-gap hot-spots should overlap spectrally with the absorption peaks, and not the film resonance.

### 4.2. Rhodamine 6G Measurements

**Figure 5:**
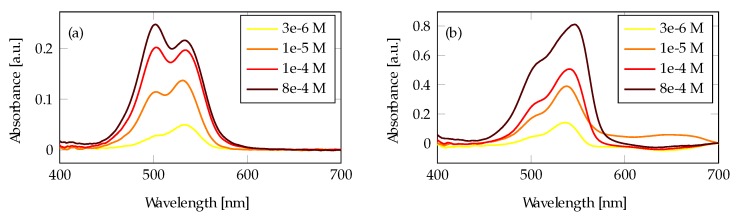
R6G measurements in a tapered fiber: (**a**) without Au film and enhancement, and (**b**) with Au film and enhancement.

The absorbance of Rhodamine 6G (R6G) measured from the tapered fibers without any Au layer is shown in Figure 5a. We first see an increase in the 530 nm absorption peak until a high concentration of over 1 × 10^−4^ mol/L is reached. This peak corresponds to the R6G monomer [34]. For concentrations above 1 × 10^−4^ mol/L, the second peak around 500 nm starts to dominate, which corresponds with the dimer peak. Figure 5b shows the absorbance in the presence of a 100 Au film on the tapered fiber: when compared to the uncoated tapered fiber, the absorbance has increased three times. Here, the monomer peak around 530 nm absorbs much more than the dimer peak at 500 nm, even at the highest concentration.

**Figure 6:**
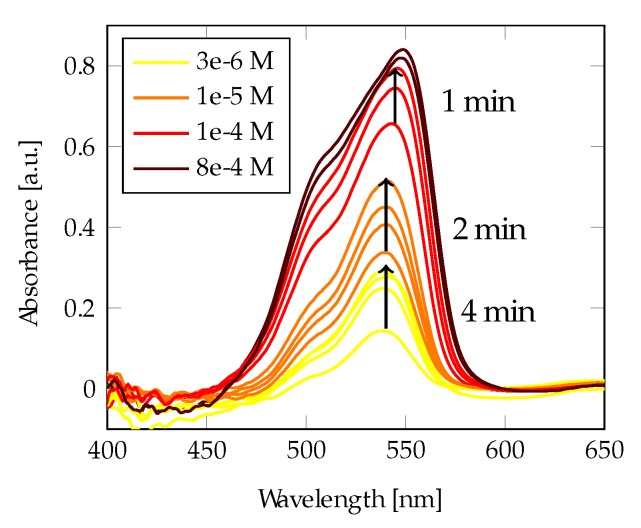
R6G measured with time from a 100 nm Au film on the taper. For each measurement, the signal increased with time until it stabilized.

The measurements shown in Figure 5a were taken immediately after sample application. However, the absorbance increased with time after the sample application and stabilized after 4 min, shown in Figure 6. This is due to the adsorption kinetics: it takes some time to achieve an equilibrium between the molecules in solution and the molecules adsorbed to the surface. The increase is most substantial for the lower concentrations, and the 8 × 10^−4^mol/L only absorbed slightly more with time. This observation corresponds to what we expect from adsorption kinetics: saturation of molecules on the surface is reached faster with a high concentration [35] than a low one. The increase in absorbance with time was much more dominant at the Au surface than on the fiber without the Au film. This effect confirms that there is an interaction between the surface and the molecules.

### 4.3. Crystal Violet Measurements

**Figure 7:**
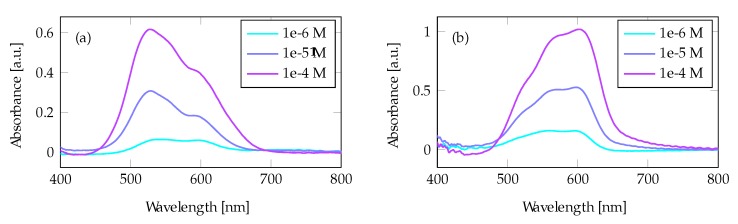
Crystal Violet (CV) measurements in a tapered fiber: (**a**) without Au film and (**b**) with a 100 nm Au film and enhancement.

The absorbance of Crystal Violet (CV) solutions using a tapered fiber without any Au deposition is shown in Figure 7a. Figure 7b shows the absorbance from a tapered fiber measured with the same CV solutions and a 100 nm Au layer. The signal is stronger than for the uncoated fiber, and the absorption spectrum is shifted towards longer wavelengths, in accordance with the plasmon resonance peak.

**Figure 8:**
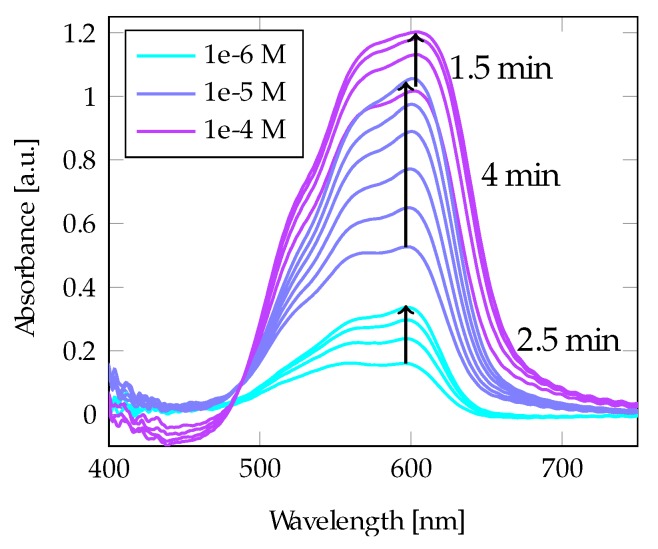
A 100 nm Au film on the taper displayed enhancement of the absorption signal of CV. The signal increased until saturation.

In line with the measurements of R6G, the CV absorbance also increased with time after application to the Au-coated fiber probe. The enhanced absorption as a function of time is seen in Figure 8. The absorbance increased by about a twofold, and the same pattern emerges as seen with R6G: the absorbance increases slightly with time until stabilization. The time varied from 1.5 to 4 min across different sample concentrations. The highest concentration (1 × 10^−4^ mol/L) stabilized faster than the other solutions. However, there was not a linear relationship between stabilization time and concentration. The most substantial increase in the signal over time was found for concentrations in the middle of the investigated concentration range, from 5 × 10^−6^ mol/L to 1 × 10^−5^ mol/L. (The same tendency was also observed for R6G but not as strongly.)

### 4.4. Comparison of the Dyes

For both the R6G and the CV measurements, the peak absorption wavelength shifted from a lower to a higher wavelength. For R6G, this is clear due to the shift in absorption from dimer to monomer peak at higher concentrations (Figure 5). CV (Figure 7) has an absorption that overlaps more with the plasmon wavelength peak than R6G and exhibits the same phenomenon. This change in absorption peak confirms the presence of enhancement, as the plasmon absorption is increased at higher wavelengths (Figure 4a). Plasmon excitations require a free-electron-like material: for wavelengths shorter than 520 nm, the electrons in an Au film can be excited within the metal, from the electronic *d* bands to the Fermi level [18,36]. Therefore, Au is generally not considered a good plasmon material at wavelengths below 520 nm, and a significant enhancement of the monomer peak at 500 nm should not be expected. The observed absorption peaks in Figure 4a at wavelengths lower than 520 nm could, therefore, be a combination of electronic band excitations and plasmons.

**Figure 9:**
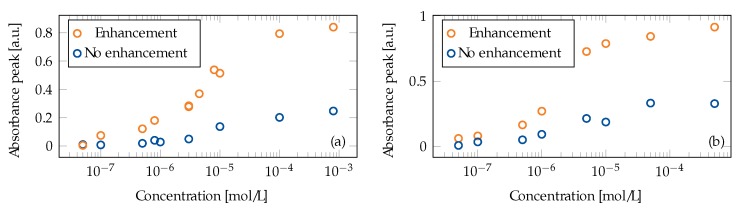
The absorption peak values for (**a**) R6G and (**b**) CV measured with the tapered fiber with and without a 100 nm Au film. The absorption signal was stabilized before it was collected.

A summary of the peak absorbance of several R6G concentrations after stabilization is shown in Figure 9a for tapers with and without the 100 nm Au film. There is an increase in all concentrations except for 5 × 10^−8^ mol/L, but the enhancement is nonlinear, as expected from adsorption isotherms that describe the molecular adsorption. For high concentrations, saturation leads to a flattening curve. The highest increase is seen for concentrations 5 × 10^−6^ mol/L to 5 × 10^−5^ mol/L.

A summary of the peak absorbance for a series of CV measurements after stabilization is plotted in Figure 9b. An increased absorption was observed at all concentrations for the Au coated fiber as compared to no Au film. A larger signal increase was seen for concentrations in the middle of the investigated range until saturation occurred at higher concentrations. However, for both dyes, the lower enhancement at low concentrations could be due to a lower SNR rather than an actual physical effect. We plan to investigate this in future studies. The saturation at higher concentration is due to the adsorption and follows a similar curve to that of a Langmuir–Freundlich isotherm [35,37].

## 5. Discussion

The tapered fibers coated with a 100 nm Au film on one side produced enhanced signal absorption for the two investigated dyes. An increase in the overall absorption was observed, as expected from the broad plasmon resonance peak overlapping with the broad absorption peak of the dyes. The absorption for both dyes increased with time, which suggests that the local field created by the plasmons contributes to and increases the absorption signal. The enhancement of 2–3 times the original absorption spectrum is in line with some reports of enhanced photoluminescence of R6G [7,38]. In this work, we have only measured the absorption enhancement of the dyes, as the subsequent fluorescence has no way to couple into the fiber.

The results are comparable to some observations of non-resonant SEIRAS in the Kretschmann configuration [39], but the enhancement is lower than other demonstrations in the infrared range [9,13]. This could be attributed to the fact that there is plasmon interaction on only part of the circumference of the taper. By using a different geometry that utilizes the full evanescent field, the enhancement could likely be increased tenfold. For certain designs of antennas and nanostructures, it has been shown that it is possible to get an increase in the plasmonic field strength with increasing wavelength [11]. A previous study on single mode fibers showed that, with doubly-deposited metal layers, the authors could tailor plasmon resonance wavelengths in the Vis/NIR for SPR refractive index sensing [21]. Leite et al. [40] demonstrated that, when the SPR peak and absorption band overlap, they can utilize the plasmon inhibition effect to make the SPR sensor specific without the use of any recognition agents. However, they did not extend this to absorption spectroscopy, as explored in our study.

The enhancement measurements shown here were all obtained from one fiber during a data collection period of two months. By following the fabrication protocol, similar results were also attained on another fiber, but with a slightly different enhancement factor. The absorbance for a tapered fiber without an Au film was measured for different tapers (following the same fabrication protocol) and was reproducible across different fibers and a period of several months.

The absorbance measurements for the dyes (Figure 9) did not follow a linear dependence with increasing concentration due to adsorption kinetics and possibly also the MM excitation (discussed in the Theory section) [23]. The measurements at high concentrations reached saturation, as has been previously reported [41]. Moreover, R6G is known to deviate from Beer’s law due to self-association dependent on concentration [34]. As this paper aims at showing enhancement of the signal and not at classifying the sensing curves, we have not provided a curve fit. For a sensing application, a rigorous calibration within the concentrations of interest should be performed. The time to reach equilibrium depends on the adsorption process. Therefore, any calibration procedure and sensor–signal interpretation must account for the resulting characteristic time constant. This is not a major complication; the same consideration applies to the more established field of SERS, where adsorption which will fit the same type of isotherm also occurs. It should also be noted that the two dyes are cations that possess a positive surface charge that adsorbs strongly to surfaces with the opposite charge, such as silica [42]. If the analyte of interest is an anion, the interaction may differ.

For future experiments, the design can be improved by first simulating the mode fields of the optical fiber and the shape of the metal nanostructures on the fiber surface to tailor the plasmon wavelength to the absorption wavelength of interest. In building a calibration model, regression methods such as principal component regression (PCR), partial least squares regression (PLSR), or artificial neural networks (ANN) may be used [43].

In this paper, we have extended and modified the technique used for SPR sensing on fibers to achieve surface-enhanced absorption spectroscopy in the visible range on fibers, as inspired by previous work on SEIRAS. The effect is demonstrated and can be developed further by patterning the surface to obtain resonances in the NIR. This could further enable remote sensing using fiber probes and label-free Vis/NIR spectroscopy by increasing the signal in targeted wavelength bands.

## Abbreviations

The following abbreviations are used in this manuscript:

Vis	visible
NIR	near-infrared
MIR	mid-infrared
SERS	surface-enhanced Raman spectroscopy
PEF	plasmon-enhanced fluorescence
SNR	signal-to-noise ratio
SEIRAS	surface-enhanced infrared absorption spectroscopy
ATR	attenuated total reflection
SPR	surface plasmon resonance
MM	multi mode
LSPR	localized surface plasmon resonance
R6G	Rhodamine 6G
CV	crystal violet
TM	Transverse Magnetic
PCR	principal component regression
PLSR	partial least squares regression
ANN	artificial neural networks
